# Stereotactical normalization with multiple templates representative of normal and Parkinson-typical reduction of striatal uptake improves the discriminative power of automatic semi-quantitative analysis in dopamine transporter SPECT

**DOI:** 10.1186/s40658-023-00544-9

**Published:** 2023-03-29

**Authors:** Ivayla Apostolova, Tassilo Schiebler, Catharina Lange, Franziska Lara Mathies, Wencke Lehnert, Susanne Klutmann, Ralph Buchert

**Affiliations:** 1 Department of Diagnostic and Interventional Radiology and Nuclear Medicine, University Medical Center Hamburg-Eppendorf, Martinistr. 52, 20246 Hamburg, Germany; 2 Department of Nuclear Medicine, Charité - Universitätsmedizin Berlin, Corporate Member of Freie Universität Berlin and Humboldt-Universität zu Berlin, Berlin, Germany

**Keywords:** Dopamine transporter, SPECT, FP-CIT, Ioflupane, Specific binding ratio, Stereotactical normalization, Template

## Abstract

**Background:**

The specific binding ratio (SBR) of ^123^I-FP-CIT in the putamen is widely used to support the interpretation of dopamine transporter (DAT) SPECT. Automatic methods for computation of the putamen SBR often include stereotactical normalization of the individual DAT-SPECT image to an anatomical standard space. This study compared using a single ^123^I-FP-CIT template image as target for stereotactical normalization versus multiple templates representative of normal and different levels of Parkinson-typical reduction of striatal ^123^I-FP-CIT uptake.

**Methods:**

1702 clinical ^123^I-FP-CIT SPECT images were stereotactically normalized (affine) to the anatomical space of the Montreal Neurological Institute (MNI) with SPM12 either using a single custom-made ^123^I-FP-CIT template representative of normal striatal uptake or using eight different templates representative of normal and different levels of Parkinson-typical reduction of striatal FP-CIT uptake with and without attenuation and scatter correction. In the latter case, SPM finds the linear combination of the multiple templates that best matches the patient’s image. The putamen SBR was obtained using hottest voxels analysis in large unilateral regions-of-interest predefined in MNI space. The histogram of the putamen SBR in the whole sample was fitted by the sum of two Gaussians. The power to differentiate between reduced and normal SBR was estimated by the effect size of the distance between the two Gaussians computed as the differences between their mean values scaled to their pooled standard deviation.

**Results:**

The effect size of the distance between the two Gaussians was 3.83 with the single template versus 3.96 with multiple templates for stereotactical normalization.

**Conclusions:**

Multiple templates representative of normal and different levels of Parkinson-typical reduction for stereotactical normalization of DAT-SPECT might provide improved separation between normal and reduced putamen SBR that could result in slightly improved power for the detection of nigrostriatal degeneration.

**Supplementary Information:**

The online version contains supplementary material available at 10.1186/s40658-023-00544-9.

## Background

Single photon emission computed tomography (SPECT) with the dopamine transporter (DAT) ligand N-ω-fluoropropyl-2β-carbomethoxy-3β-(4-I-123-iodophenyl)nortropane (^123^I-FP-CIT) is widely used for the detection (or exclusion) of nigrostriatal degeneration in clinically uncertain parkinsonian syndromes [1–5]. Visual reading of ^123^I-FP-CIT SPECT images can be complemented by the computation of specific binding ratios (SBR) to characterize ^123^I-FP-CIT binding to the DAT in the striatum and striatal subregions [6–11].

The computation of striatal SBR requires anatomical delineation of the striatum or striatal subregions and of a reference region (to estimate nondisplaceable binding) in the patient’s individual SPECT image. Automatic methods are fully reproducible and, therefore, avoid intra- and between rater variability of manual delineation [12, 13]. Automatic delineation methods often imply stereotactical normalization of the individual DAT-SPECT image from native patient space to an anatomical reference space so that standard regions-of-interest (ROIs) predefined in the anatomical reference space can be applied for the delineation. The anatomical reference space is usually represented by a template image, and the optimal transformation from individual patient space to the reference space is determined by comparing the transformed individual image with the target template image using a preselected quality metric. Stereotactical normalization based on an individual high-resolution T1-weighted magnetic resonance image (MRI) and a T1-weighted MRI as target in template space is considered the standard-of-truth for stereotactical normalization. The optimal transformation determined for stereotactical normalization of the patient’s T1-weighted MRI is applied to the patient’s DAT-SPECT image after this has been co-registered to the individual MRI. However, a recent T1-weigted MRI of sufficient quality is not always available in everyday clinical routine. In these cases, the transformation for stereotactical normalization can be estimated from the individual DAT-SPECT and a tracer-specific DAT-SPECT template [12].

Most software tools for automatic semi-quantitative analysis of DAT-SPECT use a single DAT-SPECT template representative of normal striatal ^123^I-FP-CIT uptake [12, 14–16]. This might cause a bias in favor of normal DAT-SPECT images, that is, more accurate anatomical delineation of the striatum and its subregions in case of normal ^123^I-FP-CIT uptake compared to striata with Parkinson-typical reduction of ^123^I-FP-CIT uptake. In particular, the use of an ^123^I-FP-CIT SPECT template with normal striatal uptake might cause artificial inflation and spatial shifting of striata with Parkinson-typical reduction of ^123^I-FP-CIT uptake to better match healthy striata in size and localization. This would result in overestimation of reduced putaminal SBR and, as a consequence, in reduced power to detect nigrostriatal degeneration by semi-quantitative analysis of FP-CIT SPECT.

Aim of the present study was to test for a potential benefit of simultaneously using multiple templates representative of normal and different levels of Parkinson-typical reduction of striatal ^123^I-FP-CIT uptake for stereotactical normalization compared to a single template with normal striatal signal.

## Methods

### Patients

The PACS of the Department of Nuclear Medicine of the University Medical Center Hamburg Eppendorf was searched for DAT-SPECT that had been performed to support the diagnosis of a clinically uncertain parkinsonian syndrome. The only inclusion criteria were that DAT-SPECT had been performed with low-energy-high-resolution parallel-hole or fan-beam collimators and that the raw projection data were digitally available for consistent retrospective image reconstruction. There were no further eligibility criteria to make sure that the included data were representative of everyday clinical routine at our site. This resulted in 1740 DAT-SPECT.

About two thirds of the DAT-SPECT had been included in previous studies on deep learning-based classification of DAT-SPECT [17] and data-driven identification of diagnostically useful extrastriatal signal in DAT-SPECT [18].

### SPECT imaging

SPECT had been performed between December 2008 and January 2020 according to common procedures guidelines [19, 20] with four different cameras: Siemens e.cam dual head camera equipped with low-energy-high-resolution collimators, Siemens Symbia TruePoint dual head camera with low-energy-high-resolution collimators, Siemens Symbia TruePoint with fan-beam collimators, and Mediso AnyScan Trio triple head camera equipped with low-energy-high-resolution-high-sensitivity collimators in dual head mode. Detailed acquisition parameters are given in Table 1.

**Table 1:** SPECT acquisition parameters

	Siemens e.cam with LEHR	Siemens Symbia TruePoint with LEHR	Siemens Symbia TruePoint with fan-beam	Mediso AnyScan Trio with LEHRHS (dual head mode)
# scans included in the analyses	693	144	441	424
# views	128	120	120	120
scan arc [°]	180	180	180	180
angular step [°]	2.81	3	3	3
matrix	128 × 128	128 × 128	128 × 128	128 × 128
pixel size [mm^2^]	4.80 × 4.80	3.90 × 3.90	3.90 × 3.90	2.43 × 2.43
energy window [keV]	147–171	147–171	147–171	143–175
total net scan duration [min]	32–41	30–40	40	40

All SPECT images were reconstructed retrospectively using the iterative ordered-subsets-expectation–maximization algorithm with resolution recovery implemented in the HybridRecon-Neurology tool of the Hermes SMART workstation v1.6 with parameter settings recommended for FP-CIT SPECT by Hermes (5 iterations, 15/16 subsets for 120/128 views, postfiltering with 3-dimensional Gaussian kernel of 7 mm full-width-at-half-maximum, uniform attenuation correction with narrow-beam attenuation coefficient 0.146/cm, simulation-based scatter correction, resolution recovery with a Gaussian model).

### ^123^I-FP-CIT templates

An ^123^I-FP-CIT template representative of normal striatal ^123^I-FP-CIT uptake was generated as follows: twelve DAT-SPECT images with normal striatal signal according to visual inspection were selected randomly from the 1740 DAT-SPECT included in this study. More precisely, the 1740 DAT-SPECT images were sorted in alphabetical order (according to the file names) and then the first 12 images with normal striatal signal according to visual inspection were used. Each individual image was stereotactically normalized into the anatomical space of the Montreal Neurological Institute (MNI) using the Normalize tool of the statistical parametric mapping software package (version SPM12), a custom ^123^I-FP-CIT template in MNI space generated previously [16] as target, and the following parameter settings: affine transformation (no nonlinear warping), template weighting by a binary mask of the whole head including the scalp and excluding the cerebellum predefined in MNI space, source weighting by a binary cube of 200 mm edge length centered at the center-of-mass of the individual DAT-SPECT image in patient space, MNI template regularization, preserve concentrations, voxel size 2 × 2 × 2 mm^3^, trilinear interpolation, no wrapping. Intensity normalization of the stereotactically normalized images was achieved by voxelwise scaling to the individual 75^th^ percentile of the voxel intensity in a reference region comprising the whole brain without striata, thalamus, brainstem, cerebellum, and ventricles [15]. The twelve resulting stereotactically normalized distribution volume ratio (DVR) images were averaged (soft mean). A preliminary, left–right-symmetric template was obtained by flipping the mean image at the midsagittal plane and averaging the mean and the flipped image. The final left–right-symmetric template representative of normal striatal ^123^I-FP-CIT uptake was obtained by repeating these steps two times using the preliminary template from the previous iteration as target image for stereotactical normalization.

^123^I-FP-CIT templates representative of moderate and of strong Parkinson-typical reduction of striatal uptake were generated from twelve randomly selected DAT-SPECT with moderate reduction and twelve randomly selected DAT-SPECT with strong reduction of the striatal signal. More precisely, the 1740 DAT-SPECT images were sorted in alphabetical order (according to the file names) and then the first 12 images with moderate reduction and the first 12 images with strong reduction of the striatal signal according to visual inspection were selected for the generation of the corresponding templates. The further procedure was analogous to the generation of the ‘normal’ template except that these templates were not made left–right-symmetric. For the moderate reduction template, the stereotactically normalized DVR images were left–right-flipped prior to averaging such that the striatal deficit was more pronounced in the right hemisphere in all cases. The final template representative of moderate Parkinson-typical reduction in the right hemisphere was left–right flipped at the midsagittal plane to generate a template representative of moderate Parkinson-typical reduction in the left hemisphere. This resulted in four different ^123^I-FP-CIT templates in MNI space representative of normal uptake and different stages of Parkinson-typical reduction of ^123^I-FP-CIT uptake in the striatum with attenuation and scatter correction.

**Fig. 1:**
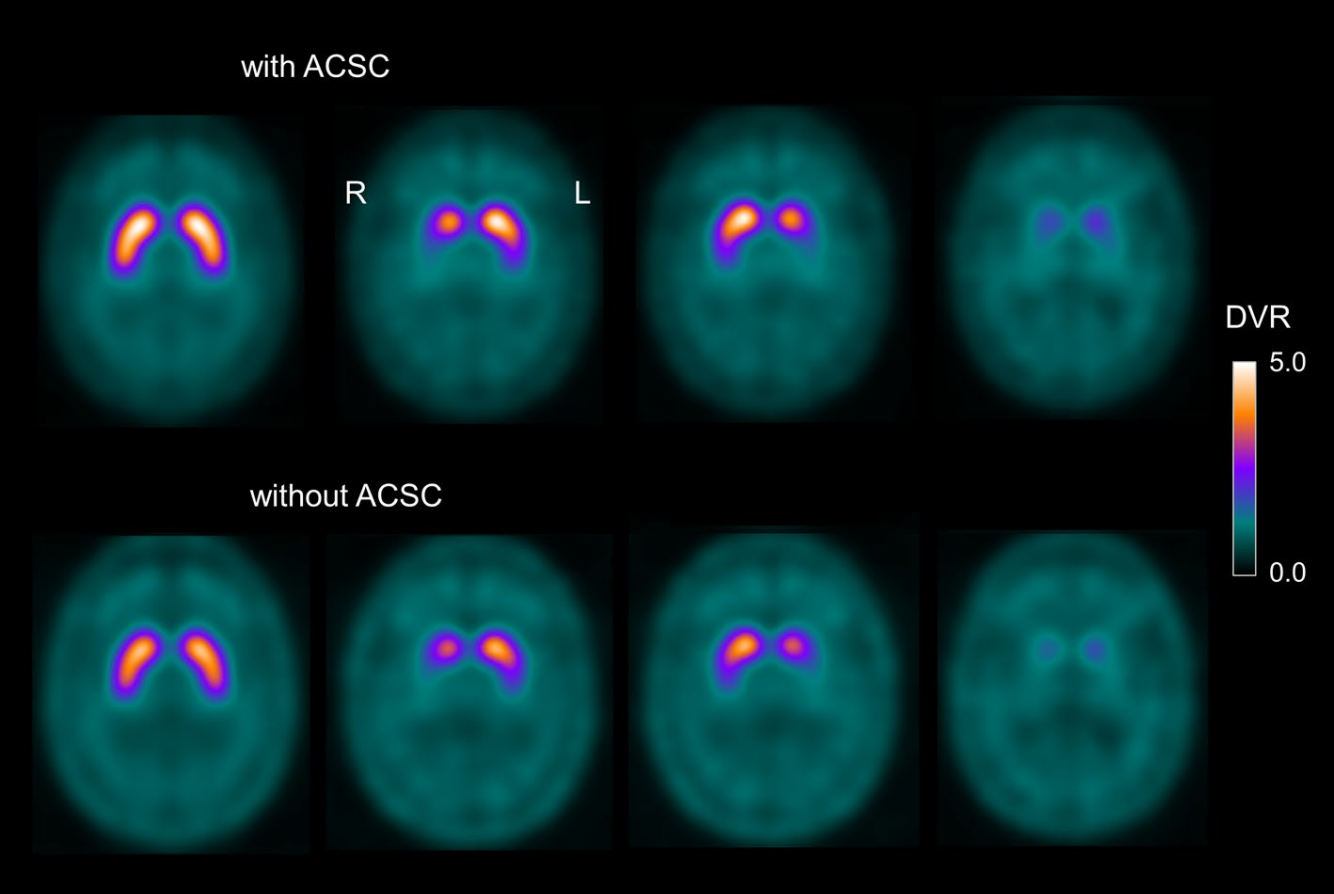
Templates of the ^123^I-FP-CIT distribution volume ratio (DVR) in MNI space. The templates are representative of normal striatal signal (left column), moderate (middle columns) and strong (last column) Parkinson-typical reduction of striatal uptake with and without attenuation and scatter correction (ACSC). Each template was generated from twelve randomly selected DAT-SPECT images. The ^123^I-FP-CIT template representative of normal striatal signal with attenuation and scatter correction (upper left) was used for single template stereotactical normalization

Four ^123^I-FP-CIT templates representative of DAT-SPECT without attenuation and scatter correction were generated from the DAT SPECT images of the same patients reconstructed from the same projection data using the iterative ordered-subsets-expectation–maximization algorithm on the Hermes SMART workstation without attenuation and scatter correction [21]. The final set of eight ^123^I-FP-CIT templates is shown in Fig. 1.

The rationale for using 12 SPECT images for each of the ^123^I-FP-CIT templates was that the SPM ^15^O-water template was created from 12 normal ^15^O-water PET images [22]. That 12 is an adequate number of images for template generation also in DAT-SPECT with ^123^I-FP-CIT was shown by Kas and co-workers who compared 4 different ^123^I-FP-CIT templates generated from 5 to 15 normal scans with respect to their impact on semi-quantitative analysis and voxel-based statistical testing [21]. They found that stereotactical normalization with these templates “provided results close enough to consider that the templates can be used interchangeably without altering the clinical interpretation” [21].

### Preprocessing of individual DAT-SPECT images

Each of the 1704 individual DAT-SPECT images (with attenuation and scatter correction) that was not used for the generation of the ^123^I-FP-CIT templates was stereotactically normalized to MNI space using either the single template representative of normal striatal ^123^I-FP-CIT uptake (with attenuation and scatter correction) as target or the set of the eight different templates. In the latter case, SPM tries to find the linear combination of these templates that best matches the intensities in the patient’s image. All other settings for stereotactical normalization and subsequent intensity scaling to obtain DVR images were as described in subsection “^123^I-FP-CIT templates”.

Stereotactical normalization was checked for major failures by visual inspection of each of the 1704 stereotactically normalized DAT-SPECT images using a display with three orthogonal views. This was done separately for the normalization results with the single template and with multiple templates.

### Semi-quantitative analysis

Two different methods were used for semi-quantitative analysis. First, the ^123^I-FP-CIT DVR in left and right putamen was estimated by hottest voxels (HV) analysis of the stereotactically normalized DVR image using large unilateral putamen masks predefined in MNI space as described previously [16]. The putamen masks were much bigger than the actual putamen volume in order to guarantee that all putaminal counts were included. The number of hottest voxels within a unilateral putamen mask to be averaged was fixed to a total volume 10 ml.

For comparison, the ^123^I-FP-CIT DVR in left and right putamen was estimated by conventional analysis, that is, by the mean of the voxel intensities in anatomical ROIs for the unilateral putamen predefined in MNI space. The unilateral putamen masks of the Automated Anatomical Labelling (AAL) atlas were used for this purpose [23].

The ^123^I-FP-CIT SBR in left and right putamen was obtained from the corresponding DVR according to SBR = DVR − 1, separately for both methods of semi-quantitative analysis. The minimum of the putamen SBR of both hemispheres was used for the further analyses. The mean putamen SBR of both hemispheres was used for comparison.

### Statistical analysis

The general linear model for repeated measures was used to test the impact of the templates (multiple templates versus single template), the ROI method (hottest voxels analysis versus anatomical AAL ROIs), and the characteristic (minimum versus mean of both hemispheres) on the SBR. The camera (e.cam with LEHR versus TruePoint with LEHR versus TruePoint with fan-beam versus AnyScan Trio with LEHRHS, Table 1) was included in the model as between-subjects factor.

The distribution of the putamen SBR was characterized by a histogram with bin width of 0.1. The resulting histogram was fitted by the sum of two Gaussians:

1 $${\text{histogram}}\,{\text{(SBR}}) = A_{1} \exp \left( { - \frac{{\left( {{\text{SBR}} - M_{1} } \right)^{2} }}{{2{\text{SD}}_{1}^{2} }}} \right) + A_{2} \exp \left( { - \frac{{\left( {{\text{SBR}} - M_{2} } \right)^{2} }}{{2{\text{SD}}_{2}^{2} }}} \right),$$

where* A*_1_, *A*_2_ are the amplitudes, *M*_1_, *M*_2_ are the mean values and SD_1_, SD_2_ are the standard deviations of the Gaussian functions. The MATLAB routine ‘fminsearch’ with default parameter settings was used for this purpose.

The power of the SBR to differentiate between normal and reduced DAT-SPECT was estimated by the effect size *d* of the distance between the two Gaussians computed as the differences between the mean values scaled to the pooled standard deviation, that is,

2 $$d = \left( {M_{2} - M_{1} } \right)/\sqrt {\frac{{{\text{SD}}_{1}^{2} + {\text{SD}}_{2}^{2} }}{2}} .$$

The cutoff *c* for differentiation between normal and reduced SBR was selected halfway between *M*_1_ and *M*_2_ in units of standard deviations, that is

3 $$c = \left( {{\text{SD}}_{2} M_{1} + {\text{SD}}_{1} M_{2} } \right)/\left( {{\text{SD}}_{1} + {\text{SD}}_{2} } \right).$$

The histogram analysis was performed separately for each combination of templates (multiple templates or single template), ROI method (hottest voxels analysis or anatomical AAL ROIs), and characteristic (minimum or mean of both hemispheres).

In order to assess the robustness of the effect size estimates, the histogram analysis was performed on 1000 random subsamples each comprising 90% of the whole DAT-SPECT sample.

The amount of stretching of individual DAT SPECT images required for stereotactical normalization was characterized by the determinant (DET) of the affine normalization transformation, separately for stereotactical normalization with multiple templates and with the single normal template. The putamen SBR was tested for association with the corresponding DET using linear regression (with constant). The DET of stereotactical normalization with multiple templates was used for the four multiple templates settings, the DET of stereotactical normalization with the single template was used for the four single template settings. The regression analysis was performed separately for DAT-SPECT with normal and reduced SBR, where the setting-specific cutoff according to Eq. (3) was used to categorize SBR as ‘normal’ or ‘reduced’.

The association between the single template DET and the multiple templates DET was tested by linear regression (without constant). The regression analysis was performed separately for DAT-SPECT with normal and reduced SBR. The regression analysis was restricted to cases in which the categorization as normal or reduced by the minimum hottest voxels SBR agreed between stereotactical normalization with the single template and stereotactical normalization with multiple templates.

Statistical analyses were conducted using SPSS version 27 (SPSS Inc., Chicago, Illinois). All p-values are given two-sided. Statistical significance was defined as *p* < 0.05.

## Results

Among the 36 cases used for template generation, the putamen SBR (multiple templates, hottest voxels analysis, minimum of both hemispheres) in the 12 images with normal striatal signal was 1.896 ± 0.313, range 1.420–2.409, corresponding to the range ≥ 65th percentile in the whole data set, it was 0.663 ± 0.148, range 0.499–1.034, corresponding to 15th–45th percentile, in the 12 images with moderate reduction, and it was 0.282 ± 0.034, range 0.228–0.333, corresponding to ≤ 5th percentile, in the 12 images with strong reduction of the striatal signal. This suggests that the range of striatal ^123^I-FP-CIT uptake encountered in clinical practice was adequately covered by the multiple templates.

There was no major failure of stereotactical normalization according to visual inspection when using multiple templates as target. There were two major failures (0.1%) with the single template as target (Additional file 1: Fig. S1). These two DAT-SPECT were excluded from the further analyses. This resulted in the inclusion of 1702 DAT-SPECT from 1675 different patients (43.1% females). The age at the time of DAT-SPECT was 66.4 ± 11.5 years (range 20–90 years). The activity dose of ^123^I-FP-CIT injected for these DAT-SPECT was 194 ± 21 MBq (range 115–291 MBq).

The general linear model for repeated measures revealed a significant between-subjects effect of the camera on the putamen SBR (*F* = 16.4, *p* < 0.0005). The mean putamen SBR was highest in scans acquired with the anyScan Trio with LEHRHS collimators and lowest in scans acquired with the e.cam with LEHR collimators. On average, putamen SBR were 0.254 larger in the images acquired with the anyScan Trio with LEHRHS collimators than in the images (from different patients) acquired with the e.cam with LEHR collimators. In addition, all tested within-subject effects on the putamen SBR were significant, including the interaction effects (templates: *F* = 1707, *p* < 0.0005; ROI: *F* = 5910, *p* < 0.0005; characteristic: *F* = 2223, *p* < 0.0005; templates*ROI: *F* = 35, *p* < 0.0005; templates * characteristic: *F* = 176, *p* < 0.0005; ROI*characteristic: *F* = 623, *p* < 0.0005; templates*ROI*characteristic: *F* = 230, *p* < 0.0005). On average, the putamen SBR was larger with the single template than with the multiple templates by 0.110 ± 0.003 (mean ± standard error of the mean), it was larger with the anatomical AAL ROIs than with the hottest voxels analysis by 0.353 ± 0.005, and it was larger when using then mean of both hemispheres compared to the minimum by 0.100 ± 0.002.

**Fig. 2:**
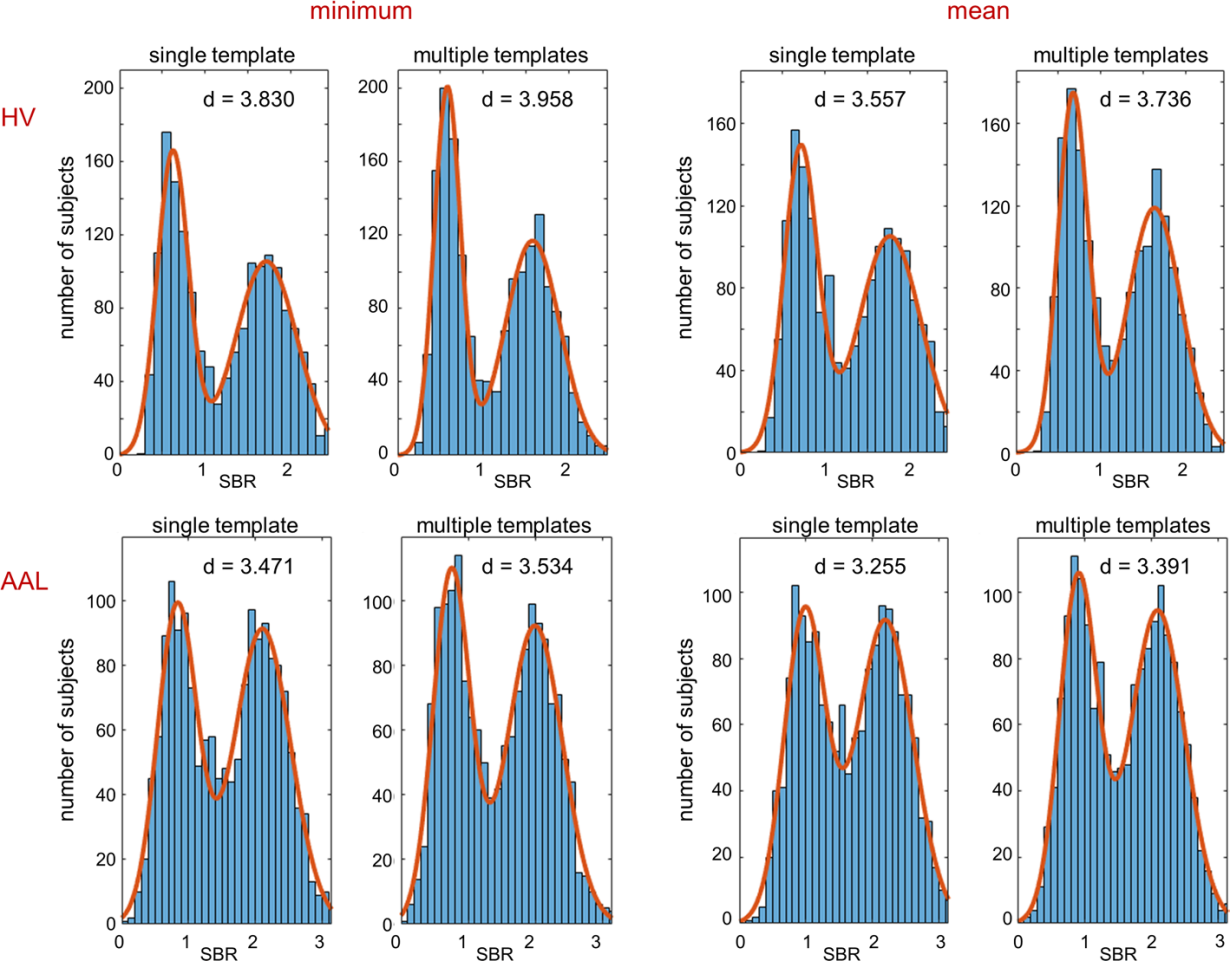
Histograms of the putamen SBR. The histograms represent the 1702 DAT-SPECT with proper stereotactical normalization according to visual inspection, separately for the different settings. The fit by the sum of two Gaussians is indicated by a continuous line. The effect size *d* of the distance between the two Gaussian functions was computed according to Eq. (2)

**Fig. 3:**
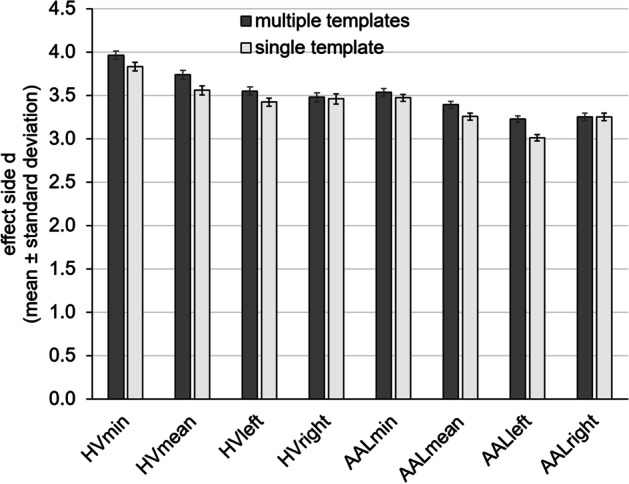
Effect size *d* of the separation between normal and reduced putamen SBR. The effect size *d* characterizes the separation of the two Gaussians from the fit of the SBR histograms (mean ± standard deviation of the effect size estimates from histogram analysis of 1000 random subsamples each comprising 90% of the whole DAT-SPECT sample)

The histograms of the putamen SBR in the included 1702 DAT-SPECT and their fit by the sum of two Gaussians are shown in Fig. 2, separately for each of the eight settings. The results of the fit (mean values, standard deviations) according to Eq. (1) and the cutoff for dichotomization of SBR according to Eq. (3) are given in Table 2. The estimates of the effect size *d* of the distance between the two Gaussians according to Eq. (2) are shown in Fig. 3. Effect size estimates were rather stable across the 1000 random 90% subsamples (coefficient of variance = standard deviation/mean ≤ 2%, Table 2).

**Table 2:** Fit of the histogram of the putamen SBR by the sum of two Gaussian functions

Single/multiple templates	Reduced		Normal		Effect size	Cutoff
	*M*_*1*_	*SD*_*1*_	*M*_*2*_	*SD*_*2*_	*d*	*c*
HV_min_	0.63/0.58	0.18/0.15	1.72/1.57	0.36/0.32	3.83 ± 0.05/3.96 ± 0.05	0.995/0.904
HV_mean_	0.71/0.67	0.20/0.18	1.77/1.63	0.37/0.32	3.56 ± 0.05/3.74 ± 0.05	1.086/1.013
HV_left_	0.73/0.67	0.22/0.19	1.78/1.63	0.37/0.33	3.42 ± 0.05/3.55 ± 0.05	1.116/1.013
HV_right_	0.66/0.61	0.20/0.17	1.73/1.58	0.39/0.36	3.46 ± 0.06/3.48 ± 0.05	1.019/0.928
AAL_min_	0.83/0.75	0.30/0.28	2.11/2.00	0.43/0.41	3.47 ± 0.04/3.53 ± 0.04	1.360/1.253
AAL_mean_	0.99/0.91	0.31/0.28	2.20/2.10	0.42/0.41	3.25 ± 0.04/3.39 ± 0.04	1.501/1.402
AAL_left_	1.07/1.00	0.35/0.34	2.25/2.21	0.43/0.41	3.01 ± 0.04/3.23 ± 0.03	1.600/1.547
AAL_right_	0.84/0.75	0.30/0.28	2.10/1.97	0.46/0.45	3.25 ± 0.04/3.25 ± 0.04	1.338/1.215

Mean values *M*_*1*_, *M*_*2*_ and standard deviations *SD*_*1*_, *SD*_*2*_ from the fit of the histogram of the putamen SBR by the sum of two Gaussian functions according to Eq. (1) in the whole sample, effect size *d* of the distance between the two Gaussian functions according to Eq. (2) (mean ± standard deviation from 1000 random 90% subsamples), and cutoff *c* for the dichotomization of SBR as normal or reduced according to Eq. (3) in the whole sample. The results are given separately for HV analysis and conventional semi-quantitative analysis with the anatomical AAL putamen masks, and for minimum (min) and mean SBR of both hemispheres and for left and right SBR. The results for stereotactical normalization with the single template or with multiples templates are given in each cell (single/multiple templates)

The cross table of binary classification of the minimum putamen SBR from hottest voxels analysis as normal or reduced with multiple templates versus single template for stereotactical normalization is given in Table 3.

**Table 3:** Binary classification of the minimum putamen SBR from hottest voxels analysis with multiple templates versus single template for stereotactical normalization

	Single template	
	Normal	Reduced
Multiple templates		
Normal	930 (54.6%)	8 (0.5%)
Reduced	29 (1.7%)	735 (43.2%)

The cutoffs derived from the fits of the two Gaussian functions were used for binary classification (0.995/0.904 for single/multiple templates)

**Fig. 4:**
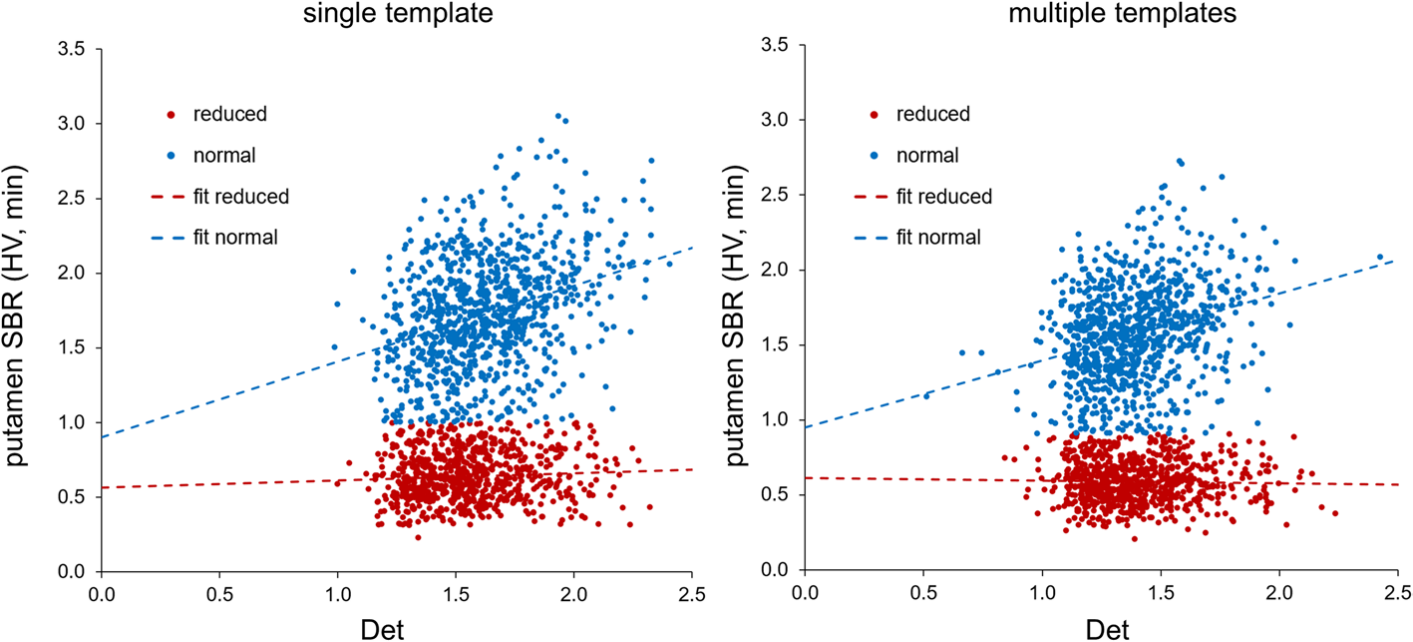
Scatter plots of the putamen SBR versus the amount of stretching by stereotactical normalization. The scatter plots of the minimum putamen SBR from hottest voxels analysis versus the DET of the affine transformation for stereotactical normalization are shown separately for single (left) and multiple (right) templates. The dashed lines indicate the result of linear regression, separately for normal and reduced SBR

Scatter plots of the minimum putamen SBR from hottest voxels analysis versus the DET of the affine transformation for stereotactical normalization are shown in Fig. 4, separately for multiple templates versus single template for stereotactical normalization. Linear regression revealed

- reduced SBR, multiple templates: SBR = 0.613 − 0.017·DET(multiple templates), *ß* = − 0.027, *p* = 0.457
- reduced SBR, single template: SBR = 0.563 + 0.049·DET(single template), *ß* = 0.070, *p* = 0.056
- normal SBR, multiple templates: SBR = 0.951 + 0.446·DET(multiple templates), *ß* = 0.291, *p* < 0.0005
- normal SBR, single template: SBR = 0.903 + 0.506·DET(single template), *ß* = 0.330, *p* < 0.0005

**Fig. 5:**
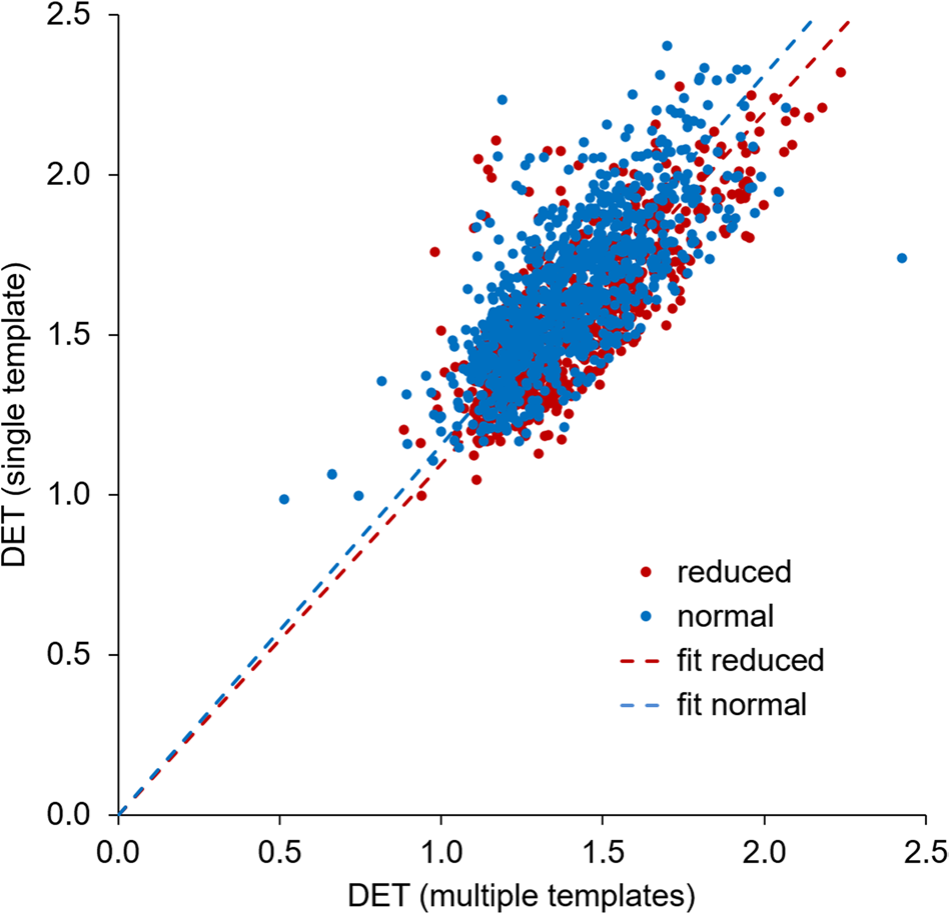
Scatter plot of the amount of stretching with the two normalization methods. The scatter plot of the DET of the affine transformation for stereotactical normalization with the single template versus DET with multiple templates includes the 1665 cases in which the binary categorization by the minimum hottest voxels SBR agreed between single template and multiple templates stereotactical normalization. The dashed lines indicate the result of linear regression, separately in the normal and in the reduced cases

Categorization as normal or reduced by the minimum hottest voxel SBR agreed between the two normalization methods in 1665 of the 1702 cases (97.8%, Cohen’s kappa = 0.956 ± 0.007, Table 3). The scatter plot of DET of the affine transformation for stereotactical normalization with the single template versus DET with multiple templates in these 1665 cases is shown in Fig. 5. Linear regression, performed separately in the normal and in the reduced cases revealed

- reduced SBR (*n* = 735): DET(single template) = 1.096·DET(multiple templates), *ß* = 0.994, *p* < 0.0005
- normal SBR (*n* = 930): DET(single template) = 1.156·DET(multiple templates), *ß* = 0.994, *p* < 0.0005

## Discussion

The primary finding of this study was that the use of multiple templates representative of normal and Parkinson-typical reduction of striatal ^123^I-FP-CIT uptake resulted in increased effect size of the difference between normal and reduced putamen SBR obtained by automatic semi-quantitative analysis compared to a single template representative of normal striatal ^123^I-FP-CIT uptake. This suggests that the power for the detection of nigrostriatal degeneration by automatic semi-quantitative analysis of DAT-SPECT might be improved by the use of multiple templates. The benefit of multiple templates versus a single template was observed for both methods of semi-quantitative analysis, hottest voxels analysis and conventional analysis with anatomical ROIs, and for both methods of combining left and right hemisphere, minimum and mean value (Fig. 3).

To some extent this can be explained by the fact that multiple templates removed the trend of a positive correlation between the putamen SBR and the determinant DET of the affine transformation for stereotactical normalization that was observed with the single normal template as target in cases with reduced SBR (standardized regression coefficient *ß* = -0.027, *p* = 0.457, with multiple templates versus *ß* = 0.070, *p* = 0.056, with the single template; Fig. 4). The DET characterizes the amount of stretching that is required to map individual DAT-SPECT to MNI space (representative of a rather large brain [24]). Thus, positive correlation of the putamen SBR with the DET suggests overestimation of the putamen SBR by overestimation of the amount of stretching resulting in artificially enlarged putamen size. This was confirmed by the finding of almost 10% larger DET in reduced cases with the single template compared to multiple templates: DET(single template) = 1.096 ⋅ DET(multiple templates).

**Fig. 6:**
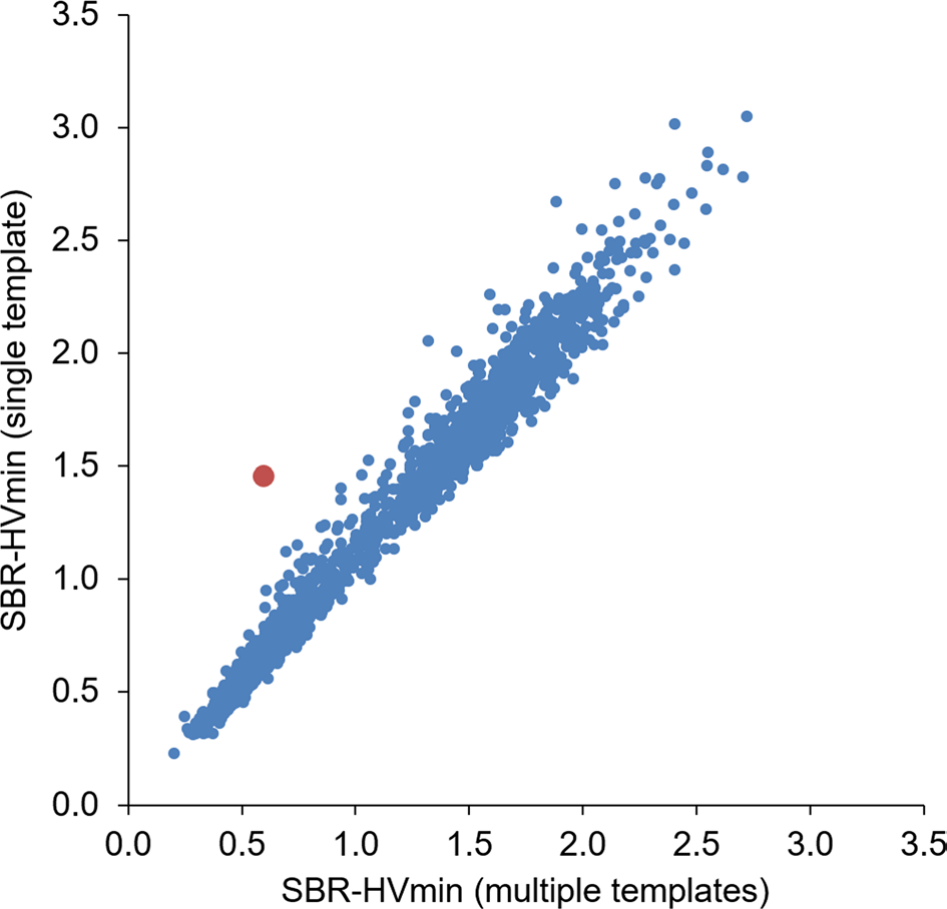
Scatter plot of the putamen SBR with the two normalization methods. The scatter plot shows the minimum hottest voxels putamen SBR obtained with the single normal template as target for stereotactical normalization versus stereotactical normalization with multiple templates. The normalized DAT-SPECT image of the outlier (red dot) is shown in Fig. 7

**Fig. 7:**
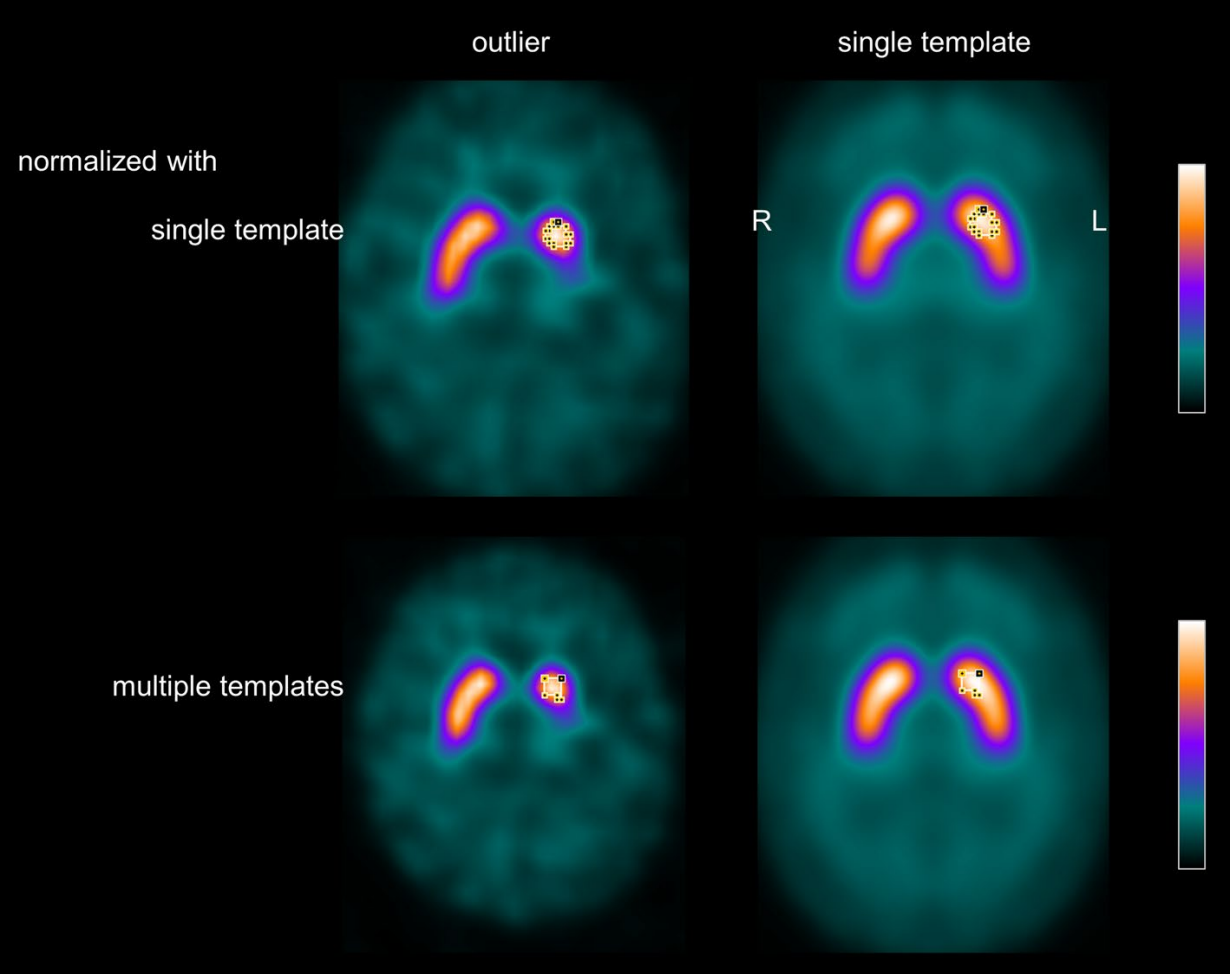
Spatial shifting of the striatum by stereotactical normalization with the single template. The figure shows a transversal image of the DAT-SPECT of the outlier (red dot in Fig. 6) after stereotactical normalization with the single normal template together with this template (top row). The 80%-isodensity contour of the outlier’s left striatum was copied to the normal template. The bottom row shows the same for the DAT-SPECT image of the outlier after stereotactical normalization with multiple templates

The scatter plot of the minimum hottest voxels putamen SBR obtained with the single normal template as target for stereotactical normalization versus stereotactical normalization with multiple templates identified one clear outlier (Fig. 6). A transversal image of the DAT-SPECT of the outlier after stereotactical normalization with the single normal template is shown in Fig. 7. The 80%-isodensity contour of the outlier’s left striatum copied to the normal template suggests that the outlier’s striatum with reduced ^123^I-FP-CIT uptake in the putamen was slightly shifted towards the posterior brain. As a consequence, part of the caudate nucleus with normal ^123^I-FP-CIT uptake ended up in the putamen mask in MNI space and resulted in strong overestimation of the putamen SBR. This effect was largely avoided by using multiple templates for stereotactical normalization including one with similar striatal signal as this patient (Fig. 7). This probably also contributed to the benefit from the multiple templates with respect to the differentiation of normal and reduced putamen SBR.

However, the impact of the stereotactical normalization on binary categorization of DAT-SPECT as normal or reduced was rather small. Binary categorization by the minimum hottest voxels putamen SBR with multiple templates or the single normal template for stereotactical normalization were concordant in 1665 of 1702 cases (97.8%, Table 3). Among the 37 cases with discrepant categorization (2.2%), there were almost 4 times more cases (*n* = 29) that were categorized as reduced with multiple templates and as normal with the single template than the other way around, that is, cases that were categorized as normal with multiple templates and as reduced with the single template (*n* = 8). Clinical follow-up o*f* ≥ 12 months was available in 10 of the 37 discrepant cases. In all these 10 cases the patients were diagnosed with a neurodegenerative etiology of the parkinsonian syndrome at follow-up (Parkinson’s disease: *n* = 8, parkinsonian type multiple system atrophy: *n* = 1, unspecified: *n* = 1). Binary categorization of the minimum hottest voxels putamen SBR with the single normal template was true positive/false negative in 3/7 of these 10 cases, binary categorization of the putamen SBR with the multiple templates was true positive/false negative in 7/3 of the 10 cases. The SPECT images of the 37 discrepant cases were retrospectively assessed visually by three independent readers. Relative to the majority read of the three readers, the multiple templates approach resulted in 3 false positive and 7 false negative cases, the single normal template approach resulted in 1 false positive and 26 false negative cases. Together these findings suggest that potential improvement of discriminative power by multiple templates might be mainly driven by improved sensitivity for the detection of nigrostriatal degeneration.

The fact that the impact of multiple templates on the binary categorization of DAT-SPECT was rather small, to some extent might be due to a ceiling effect: any reasonable method for semi-quantitative DAT-SPECT analysis provides relatively high diagnostic accuracy that leaves room for small improvement only [8]. This is explained by the rather high symptom threshold in neurodegenerative Parkinsonian syndromes. Post-mortem studies have shown that motor symptoms in Parkinson’s disease start at rather advanced stages of nigrostriatal degeneration when loss of DAT in the unilateral putamen has reached about 50% [25]. However, small improvement of the discriminative power of semi-quantitative analysis in DAT-SPECT that might be achieved by the use of multiple templates for stereotactical normalization comes practically at no costs. In particular, additional procedures (e.g., blood sampling) or prolonged scan duration (e.g., for dynamic imaging) are not required. The multiple templates have to be generated only once. The multiple templates generated for this study are freely available on request.

An additional (small) benefit from the use of multiple templates was the complete lack of normalization failures with multiple templates. There was a very small proportion (2 of 1704, 0.1%) of normalization failures with the single template. These failures most likely were associated with rather prominent ^123^I-FP-CIT uptake in the salivary glands (Additional file 1: Fig. S1), since the failure could be avoided by manual cropping of the salivary glands prior to stereotactical normalization in both cases. However, visual quality check of stereotactical normalization is mandatory in automatic semi-quantitative analysis independent of the template(s) used as target for stereotactical normalization.

Among the 1665 cases with concordant categorization, 55.9% were categorized as normal, 44.1% as reduced. This is in line with the recommendation to restrict DAT-SPECT to clinically uncertain cases, as this recommendation implies an about 50% pre-test probability of nigrostriatal degeneration.

There was a rather strong positive correlation between the putamen SBR and the determinant DET of the affine transformation for stereotactical normalization in normal DAT-SPECT. This was observed with the single template and with multiple templates as target for stereotactical normalization (Fig. 3), although the correlation was slightly weaker with multiple templates (*ß* = 0.291 versus *ß* = 0.330, both *p* < 0.0005). Linear regression of the minimum putamen SBR on DET performed separately for each of the 4 different cameras (Table 1) confirmed this unexpected finding. We rather expected a negative correlation between the putamen SBR and DET, based on the following rationale: larger DET indicates a smaller brain in native patient space and, therefore, more pronounced underestimation of the actual putamen SBR due to partial volume effects. The observed positive correlation between the putamen SBR and the DET in the normal DAT-SPECT is not specific for the hottest voxels method used for semi-quantitative analysis, since the same correlation was observed with the conventional semi-quantitative analysis using AAL putamen masks (results not shown). We hypothesize that the positive correlation between the putamen SBR and the DET was driven by residual variability of spatial resolution in the DAT-SPECT images despite harmonized image reconstruction, at least to some extent (Additional file 2: Fig. S2).

The use of multiple templates for stereotactical normalization has proven beneficial also in other nuclear brain imaging procedures, e.g., positron emission tomography with FDG [22, 26, 27] or ligands for senile amyloid plaques [28]. For DAT-SPECT, bias by the template might also be avoided by using a single template representing the mean image of an equal number of normal and reduced scans [29].

The set of templates used for the multiple templates approach in the current study combined templates with attenuation and scatter correction and templates without attenuation and scatter correction. The rationale for this was that there is considerable between-subjects variability of the ^123^I-FP-CIT uptake in the scalp. DAT-SPECT images without attenuation and scatter correction show more prominent signal in the scalp compared to DAT-SPECT images with attenuation and scatter correction (Fig. 1). Thus, inclusion of the templates without attenuation and scatter correction resulted in increased variability of the scalp signal among the multiple templates. This was expected to allow better modeling of the DAT-SPECT images of individual patients by linear combinations of the multiple templates. Furthermore, there is no risk of overfitting here so that additional templates in general are expected to improve the performance of the multiple templates approach.

The ^123^I-FP-CIT template representative of normal striatal ^123^I-FP-CIT uptake was made left–right-symmetric by left–right flipping and then averaging the original and the flipped image. The ^123^I-FP-CIT template representative of moderate Parkinson-typical reduction in the left hemisphere was obtained by left–right flipping of the ^123^I-FP-CIT template representative of moderate Parkinson-typical reduction in the right hemisphere. The rationale for this was to eliminate left–right asymmetry in the template(s) that might cause artificial left–right-asymmetry in SBR estimates.

Secondary findings of this study were that (i) hottest voxels analysis outperformed conventional semi-quantitative analysis with anatomical putamen ROIs predefined in template space and (ii) the minimum of the putamen SBR of both hemispheres outperformed left and right SBR as well as the mean of both hemispheres with respect to the differentiation of normal and reduced SBR (Table 2, Fig. 3), in line with previous studies [14, 30].

Another secondary finding of this study was the significant between-subjects effect of the camera on the putamen SBR despite the fact that all SPECT images were reconstructed retrospectively using the same iterative ordered-subsets-expectation–maximization algorithm with resolution recovery in order to minimize variability of no interest associated with camera- and/or collimator-specific effects. The mean putamen SBR was highest in scans acquired with the anyScan Trio with LEHRHS collimators and lowest in scans acquired with the e.cam with LEHR collimators. This might be due to differences in the patient samples from the two cameras. However, the difference in pixel size of the projection data between the two cameras might also have contributed (2.43 versus 4.80 mm edge length, Table 1).

Finally, this study proposes the effect size of the distance between two Gaussians fitted to the histogram of the putamen SBR in a patient sample as clinically relevant quality metric. This metric requires a sufficiently large sample size so that the shape of the histogram is sufficiently smooth and stable with respect to the choice of histogram bins. In this study, 1702 DAT-SPECT were included in the analysis, which proved sufficient.

The following limitations of this study should be noted. First, there was no standard-of-truth diagnosis available, e.g., clinical diagnosis by a movement disorder specialist ≥ 2 years after DAT-SPECT. Thus, the impact of single versus multiple templates for stereotactical normalization on the performance of the putamen SBR to differentiate between neurodegenerative and non-neurodegenerative parkinsonian syndromes could not be assessed by conventional performance measures such as sensitivity and specificity. Second, the templates were generated from images reconstructed with the same iterative algorithm as the individual DAT-SPECT used for the performance testing. The impact of different reconstruction algorithms in non-harmonized settings was not tested. Third, the impact of single versus multiple templates was not tested in DAT-SPECT with atypical reduction of the striatal signal, e.g., most pronounced reduction in the caudate nucleus (e.g., due to a vascular/structural lesion).

## Conclusions

Multiple templates representative of normal and different levels of Parkinson-typical reduction for stereotactical normalization of DAT-SPECT might provide slightly improved separation between normal and reduced putamen SBR that could result in improved power for the detection of nigrostriatal degeneration.

## Supplementary Information

**Additional file 1: Fig. S1**. Failure of stereotactical normalization with the single template occurring in two of the 1704 DAT-SPECT.

**Additional file 2: Fig. S2**. Impact of the spatial resolution in the reconstructed DAT-SPECT image on the relationship between the putamen SBR and the amount of stretching for stereotactical normalization.

## Data Availability

The template images generated during this study are available on request.

## Abbreviations

^123^I-FP-CIT: N-ω-fluoropropyl-2β-carbomethoxy-3β-(4-I-123-iodophenyl)nortropane
*A*: Amplitude
AAL: Automated Anatomical Labelling
ACSC: Attenuation and scatter correction
*ß*: Standardized regression coefficient
*c*: Cutoff
*d*: Effect size
DAT: Dopamine transporter
DET: Determinant (of affine transformation to MNI space)
DVR: Distribution volume ratio
HV: Hottest voxels
*M*: Mean value
MNI: Montreal Neurological Institute
MRI: Magnetic resonance imaging
ROI: Region-of-interest
SBR: Specific binding ratio
*SD*: Standard deviation
SPECT: Single photon emission computed tomography
SPM: Statistical parametric mapping
