# Supplementary material for: Stereotactical normalization with multiple templates representative of normal and Parkinson-typical reduction of striatal uptake improves the discriminative power of automatic semi-quantitative analysis in dopamine transporter SPECT
Source: EJNMMI Phys. 2023 Mar 29;10:25. doi: 10.1186/s40658-023-00544-9 (PMC10060476; doi:10.1186/s40658-023-00544-9)
Supplement: Supplementary file 1 — Additional file 1: Fig. S1. Failure of stereotactical normalization with the single template occurring in two of the 1704 DAT-SPECT. [file 40658_2023_544_MOESM1_ESM.docx]

**Online supplementary**

**Stereotactical normalization with multiple templates representative of normal and Parkinson-typical reduction of striatal uptake improves the discriminative power of automatic semi-quantitative analysis in dopamine transporter SPECT**

Ivayla Apostolova^1*^, Tassilo Schiebler^1*^, Catharina Lange^2^, Franziska Mathies^1^, Wencke Lehnert^1^, Susanne Klutmann^1^, Ralph Buchert^1^

^1^Department of Diagnostic and Interventional Radiology and Nuclear Medicine, University Medical Center Hamburg-Eppendorf, Hamburg, Germany

^2^Department of Nuclear Medicine, Charité - Universitätsmedizin Berlin, Corporate Member of Freie Universität Berlin and Humboldt-Universität zu Berlin, Berlin, Germany

^*^The first two authors contributed equally

**
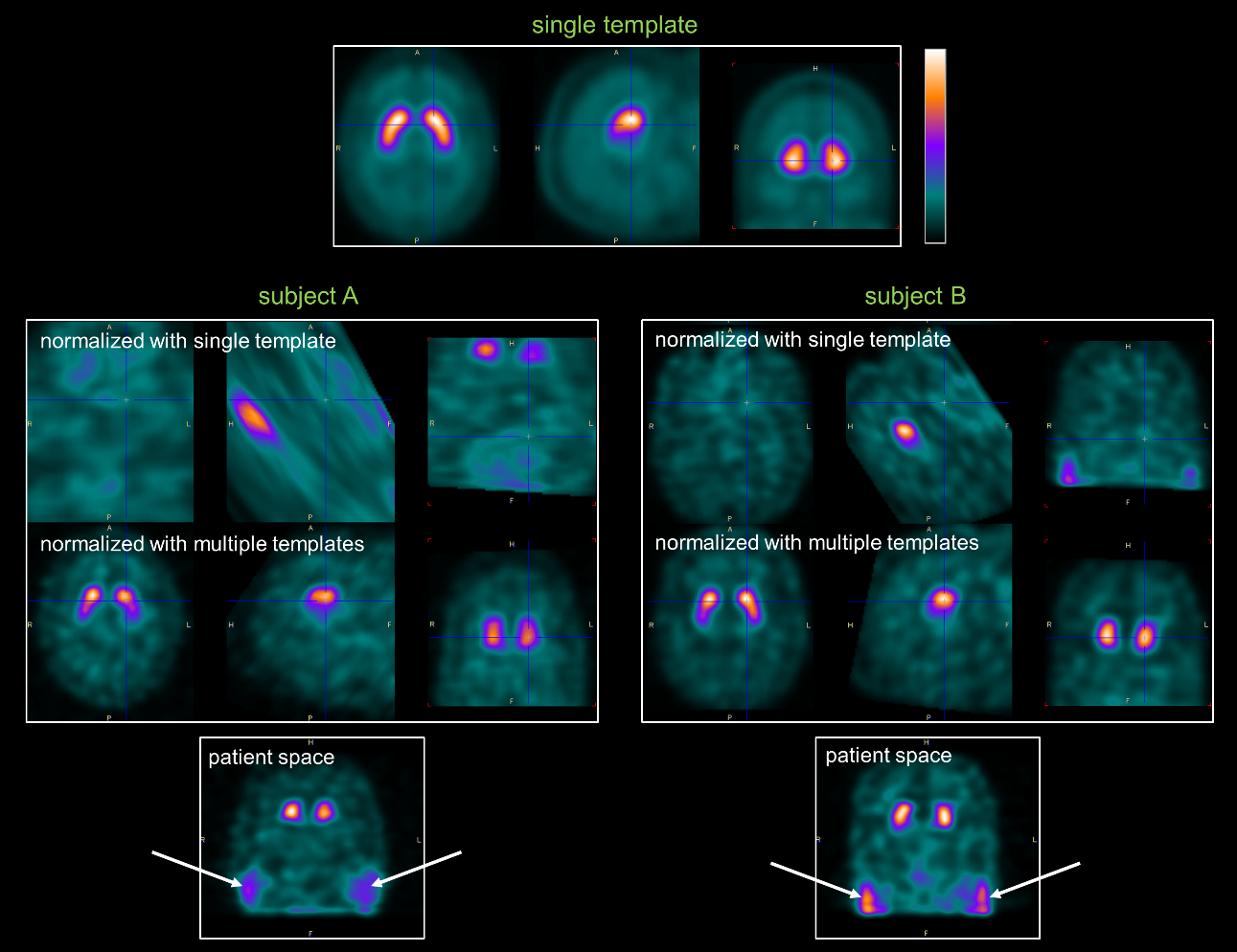
**

**Supplementary Figure S1** Failure of stereotactical normalization with the single template occurred in two of the 1704 DAT-SPECT (subjects A, B). Stereotactical normalization with multiple templates worked properly in both cases. Stereotactical normalization with the single template also worked properly when the salivary glands (arrows) were cropped from the DAT-SPECT image in patient space prior to stereotactical normalization
