# Supplementary material for: Stereotactical normalization with multiple templates representative of normal and Parkinson-typical reduction of striatal uptake improves the discriminative power of automatic semi-quantitative analysis in dopamine transporter SPECT
Source: EJNMMI Phys. 2023 Mar 29;10:25. doi: 10.1186/s40658-023-00544-9 (PMC10060476; doi:10.1186/s40658-023-00544-9)
Supplement: Supplementary file 2 — Additional file 2: Fig. S2. Impact of the spatial resolution in the reconstructed DAT-SPECT image on the relationship between the putamen SBR and the amount of stretching for stereotactical normalization. [file 40658_2023_544_MOESM2_ESM.docx]

**Online supplementary**

**Stereotactical normalization with multiple templates representative of normal and Parkinson-typical reduction of striatal uptake improves the discriminative power of automatic semi-quantitative analysis in dopamine transporter SPECT**

Ivayla Apostolova^1*^, Tassilo Schiebler^1*^, Catharina Lange^2^, Franziska Mathies^1^, Wencke Lehnert^1^, Susanne Klutmann^1^, Ralph Buchert^1^

^1^Department of Diagnostic and Interventional Radiology and Nuclear Medicine, University Medical Center Hamburg-Eppendorf, Hamburg, Germany

^2^Department of Nuclear Medicine, Charité - Universitätsmedizin Berlin, Corporate Member of Freie Universität Berlin and Humboldt-Universität zu Berlin, Berlin, Germany

^*^The first two authors contributed equally


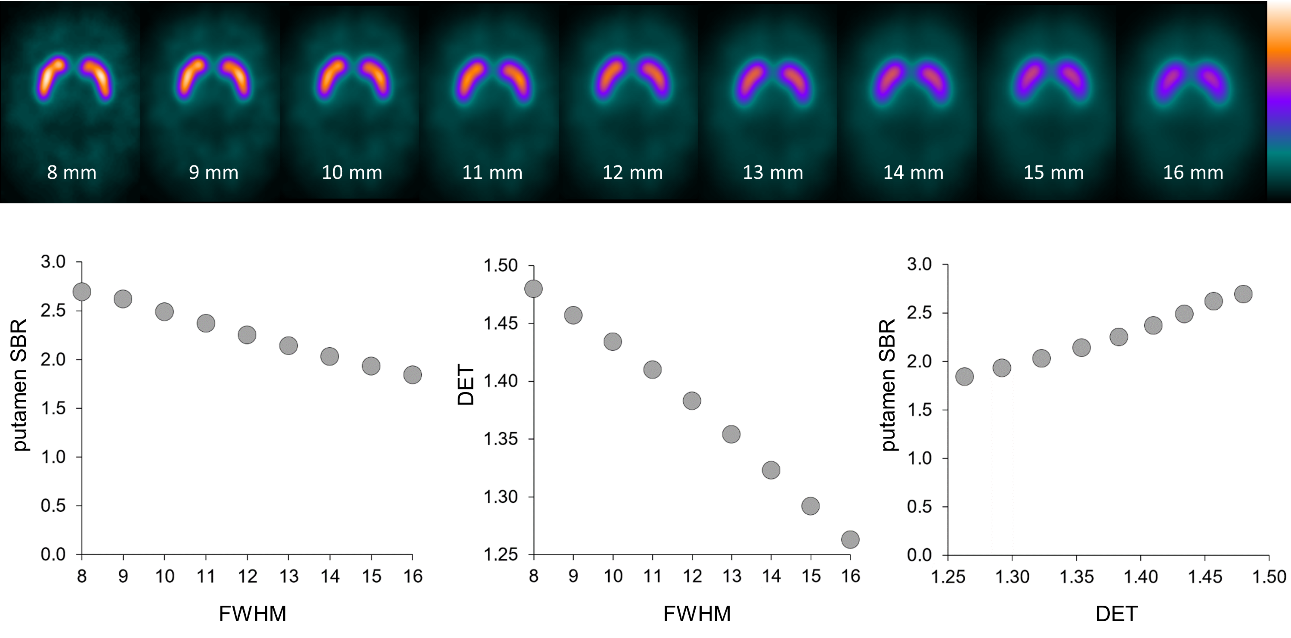


**Supplementary Figure S2** A high-resolution DAT-SPECT image (about 8 mm full-width-at-half-maximum, FWHM) acquired with the AnyScan Trio triple head SPECT camera equipped with multiple pinhole collimators specifically designed for DAT-SPECT [1] was smoothed to 9, 10, ..., 16 mm FWHM by convolution of the original DAT-SPECT image in native patient space with isotropic Gaussian kernels. The resulting DAT-SPECT were stereotactically normalized with multiple templates. A transversal slice of the stereotactically normalized images is shown in the upper part of the figure. The minimum of the hottest voxels putamen SBR of both hemispheres and the determinant (DET) of the affine transformation to template space was determined for each image. Scatter plots of the putamen SBR and of the DET versus the FWHM, and of the putamen SBR versus the DET are shown in the lower part of the figure. The putamen SBR was positively correlated with the DET. This suggests that the positive correlation between the putamen SBR and the DET observed in the normal DAT-SPECT included in this study (Figure 4) might have been driven by residual variability of spatial resolution in the DAT-SPECT images despite harmonized image reconstruction, at least to some extent.

1. Tecklenburg K, Forgacs A, Apostolova I, Lehnert W, Klutmann S, Csirik J, et al. Performance evaluation of a novel multi-pinhole collimator for dopamine transporter SPECT. Physics in Medicine and Biology. 2020;65. doi:ARTN 16501510.1088/1361-6560/ab9067.
